# Intestinal Flora and Myocarditis: Potential Mechanisms and Therapeutic Strategies Affecting Disease Progression and Cardiac Function

**DOI:** 10.3390/ijms27156706

**Published:** 2026-07-27

**Authors:** Qianyi Liu, Dan Huang, Kun Huang, Zhaohui Wang

**Affiliations:** 1 Department of Cardiology, Union Hospital, Tongji Medical College, Huazhong University of Science and Technology, Wuhan 430022, China; 2 Hubei Key Laboratory of Biological Targeted Therapy, Union Hospital, Tongji Medical College, Huazhong University of Science and Technology, Wuhan 430022, China; 3 Hubei Provincial Engineering Research Center of Immunological Diagnosis and Therapy for Cardiovascular Diseases, Union Hospital, Tongji Medical College, Huazhong University of Science and Technology, Wuhan 430022, China; 4 Key Laboratory of Biological Targeted Therapy, Huazhong University of Science and Technology, Ministry of Education, Wuhan 430022, China

**Keywords:** myocarditis, gut flora, metabolites, therapy

## Abstract

Myocarditis is a clinically challenging form of inflammatory heart disease with heterogeneous etiologies, limited diagnostic tools, no targeted therapies, and a substantial risk of progression to heart failure or sudden cardiac death, particularly in young adults. Emerging evidence has increasingly associated myocarditis with gut microbiota dysbiosis. This review explores the gut–myocarditis axis, highlighting key mechanisms and therapeutic strategies. Significant alterations in gut microbial composition are observed in myocarditis patients and animal models. Gut microbiota influences disease development through multiple pathways: compromised intestinal barrier integrity leading to bacterial translocation and systemic inflammation via MAMP/PRR signaling (e.g., TLRs, NLRs); production of metabolites—including pro-inflammatory trimethylamine N-oxide (TMAO), anti-inflammatory short-chain fatty acids (SCFAs), and immunomodulatory bile acids—that regulate host inflammatory responses, immune cell differentiation, oxidative stress, and fibrotic remodeling; and molecular mimicry, where microbial peptides (e.g., from Bacteroides thetaiotaomicron) trigger cross-reactive autoimmune responses against cardiac proteins. Regarding therapeutic strategies, this review discusses fecal microbiota transplantation (FMT), probiotics, prebiotics, dietary modulation, and emerging approaches including engineered bacteria and oral nanomedicines. Although these strategies hold promise, their efficacy and safety remain to be validated in large-scale clinical trials, and further investigation is warranted.

## 1. Introduction

Myocarditis is an inflammation disorder of the cardiac muscle that can occur across all age groups. This condition induces degeneration, necrosis, and fibrosis of cardiomyocytes, potentially impairing both systolic and diastolic functions. In severe cases, it may progress to heart failure, arrhythmias, or sudden cardiac death [1,2]. The true incidence of myocarditis is likely substantially underestimated due to the absence of reliable early diagnostic methods. Persistent myocarditis can evolve into inflammatory cardiomyopathy, characterized by chronic inflammation-mediated cardiac remodeling and dysfunction, where immune cells, cytokines, and autoantibodies play pivotal roles in disease pathogenesis [3]. The etiology of myocarditis encompasses both infectious and non-infectious causes. Infectious agents include viruses, which are the most prevalent cause, as well as bacteria, fungi, and parasites. Non-infectious factors, on the other hand, comprise autoimmune disorders, allergic reactions, and chemical- or drug-induced cardiotoxicity. These pathogenic factors trigger myocardial inflammation and injury, with subsequent tissue remodeling and fibrosis potentially exacerbating cardiac dysfunction [4].

The intestinal microbiota represents one of the most complex and diverse microbial ecosystems in the human body, having co-evolved with the host over millennia to establish a sophisticated symbiotic relationship [5]. Under physiological conditions, a dynamic equilibrium exists between the host and gut micro-organisms. The commensal gut flora not only serves as a biological barrier for the intestinal mucosa but also ferments indigestible dietary fibers, generating beneficial metabolites such as short-chain fatty acids (SCFAs). These metabolites provide energy for intestinal epithelial cells while exerting anti-inflammatory effects and preserving intestinal barrier integrity.

**Figure 1:**
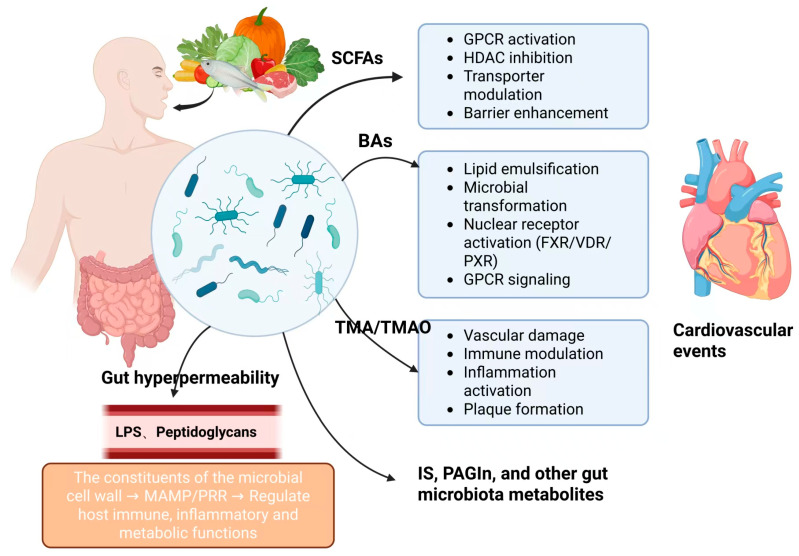
Model of gut–heart axis hypothesis. Gut microbiota is involved in the pathogenesis of cardiovascular diseases via direct or indirect mechanisms. Gut microbiota-associated pathologies (GMAPs), such as gut hyperpermeability, facilitate the translocation of microbial cell wall components (e.g., LPS, peptidoglycans), which act as microbe-associated molecular patterns (MAMPs) and engage pattern recognition receptors (PRRs) to modulate host immune, inflammatory, and metabolic responses. Short-chain fatty acids (SCFAs) exert protective effects through G protein-coupled receptor (GPCR) activation, histone deacetylase (HDAC) inhibition, transporter modulation, and enhancement of the gut barrier. Bile acids (BAs) contribute to lipid emulsification, microbial transformation, nuclear receptor activation (e.g., FXR, VDR, PXR), and GPCR signaling. Trimethylamine (TMA) and its derivative trimethylamine N-oxide (TMAO) promote vascular damage, immune dysregulation, inflammation, and plaque formation, ultimately contributing to cardiovascular events. Additional gut microbiota metabolites, including indoxyl sulfate (IS) and p-cresyl glucuronide (PAGIn), further exacerbate cardiovascular risk. This model underscores the multifaceted interplay between gut microbial metabolism and cardiovascular pathophysiology. Created in BioRender. Liu, Q. (2026) https://BioRender.com/57oad9w.

Furthermore, the gut microbiota interacts with gut-associated lymphoid tissue (GALT), playing a crucial role in immune system development and homeostasis [6,7]. Disruption of this balance—termed dysbiosis—can impair gut function and influence extra-intestinal organs via the “gut–X axis.” The “gut–cardiac axis” hypothesis posits that gut microbial imbalance may contribute to cardiovascular diseases (e.g., atherosclerosis, hypertension, and heart failure) through mechanisms involving systemic inflammation, immune dysregulation, and gut-derived metabolites [8,9] (Figure 1).

Despite advances in immunosuppressive and supportive therapies, the clinical management of myocarditis remains challenging, with no targeted therapies currently available and a subset of patients progressing to heart failure or requiring cardiac transplantation. In this context, the gut–heart axis has emerged as a promising yet underexplored avenue. Based on previous studies, this review further elucidates the pathophysiological role of the gut microbiota in myocarditis onset and progression, and explores associated therapeutic strategies. It reviews the multi-specific recognition of microbial components by pattern recognition receptors (PRRs) and elaborates on the role of translocated intestinal microbial DNA and microbial metabolites in cardiovascular diseases. Therapeutically, the review proposes building upon traditional treatment methods by utilizing drug microbiomics to guide personalized medication and developing precise intervention strategies, such as genetically engineered bacteria and oral nanomedicines.

## 2. Characterization of the Intestinal Flora in the State of Myocarditis

Clinical studies have revealed significant alterations in gut microbiota composition in myocarditis patients compared to healthy controls. Notably, enterovirus-reactive antibodies and nucleic acids have been detected in approximately 70% of dilated cardiomyopathy (DCM) cases. Leveraging large-scale GWAS data, a Mendelian randomization study encompassing over 420,000 participants (including 633 myocarditis cases) identified 14 gut microbial taxa with causal relationships to myocarditis risk. Specifically, *Clostridia*, *Ruminococcaceae*, and *Bilophila* were associated with reduced risk, whereas *Lactobacillales* and *Clostridium difficile* were associated with increased risk [10]. Mendelian randomization analysis demonstrated that a 1-unit increase in *Escherichia*/*Shigella* group concentration elevates myocarditis risk by 38.1% and hypertrophic cardiomyopathy risk by 13.3% [11]. Experimental autoimmune myocarditis (EAM) mice exhibit increased gut microbial diversity and *Firmicutes*/*Bacteroidetes* (F/B) ratio, due to decreased *Bacteroidetes* and/or increased *Firmicutes* abundance [12].

Notably, gut commensal-derived mimetic peptides can induce inflammatory cardiomyopathy in genetically predisposed individuals. Patients with cardiomyopathy show enhanced *Bacteroides*-specific CD4+ T cell and B cell responses. Specifically, the intestinal bacterium *Bacteroidetes thetaiotaomicron* (*B. theta*) produces peptides structurally similar to the cardiac protein MYH6, called mimetic peptides, which are recognized by cardiac-specific CD4 T cells and trigger a cross-immune response [13]. Activated cardiac-specific CD4 T cells release cytokines such as IL-17 and IFN-γ, which attract immune cells such as macrophages and neutrophils to cardiac tissues and trigger an inflammatory response.

While comprehensive understanding of myocarditis pathophysiology remains incomplete, dysregulated immune responses, inflammation, and autoantibody production significantly contribute to disease progression. Myocardial contractile dysfunction develops alongside impaired cardiomyocyte metabolism and cell death, with subsequent remodeling and fibrosis worsening cardiac function and prognosis. Although gut–myocarditis interactions require further elucidation, emerging evidence links intestinal permeability and microbial metabolites to these cardiovascular pathological changes.

## 3. Potential Mechanisms of Gut Microbiota Involvement in the Development of Myocarditis

**Figure 2:**
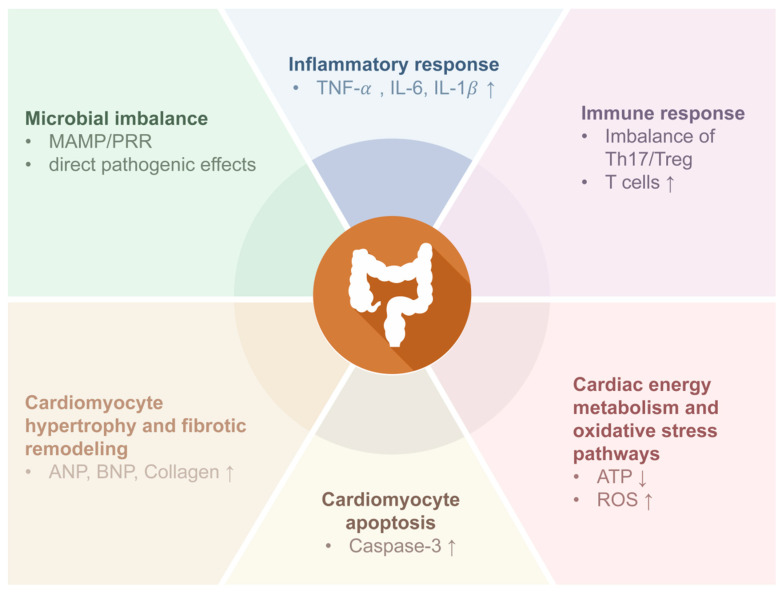
Potential mechanisms of gut microbiota involvement in the development of myocarditis. The gut microbiota contributes to myocarditis pathogenesis by compromising intestinal barrier integrity and producing microbiota-derived metabolites. These metabolites modulate disease progression through five interconnected mechanisms: regulating inflammatory responses, mediating immune imbalance, disrupting cardiac energy metabolism and inducing oxidative stress, promoting cardiomyocyte hypertrophy and fibrotic remodeling, and inducing cardiomyocyte apoptosis. Arrows indicate the direction of change (↑: increase/upregulation; ↓: decrease/downregulation). Figure 2 was generated using Microsoft PowerPoint (version 12.1.0.26375).

Gut microbiota may contribute to myocarditis pathogenesis by modulating disease mechanisms (Figure 2). Elucidating the link between gut microbial composition and cardiac dysfunction could enhance our understanding of host–microbe interactions and reveal novel therapeutic targets.

### 3.1. Gut Microbes Influence the Intestinal Barrier Involved in Disease Development

The gut serves as a primary entry point for numerous pathogens, with the intestinal barrier playing a pivotal role in maintaining systemic health. Dysbiosis compromises intestinal barrier integrity, increasing epithelial permeability and facilitating bacterial translocation into systemic circulation. This process engages innate immunity through MAMP/PRR signaling pathways [14], which have been implicated in both cardiac pathology and chronic infections. Micro-organisms produce various pathogen-associated molecular patterns (PAMPs) recognized by pattern recognition receptors (PRRs) including membrane-bound Toll-like receptors (TLRs) and cytosolic NOD-like receptors (NLRs, including NOD1 and NOD2) [15,16]. These interactions trigger intracellular signaling cascades (TRIF/MyD88 pathways), culminating in effector molecule secretion and immune activation [17,18,19].

Intestinal barrier dysfunction is a key determinant of microbial translocation and systemic inflammation. Clinically, several biomarkers have been established to assess intestinal barrier integrity and endotoxin exposure. Lipopolysaccharide (LPS) is a major component of Gram-negative bacterial outer membranes and serves as a direct marker of bacterial translocation when detected in circulation; elevated LPS levels have been associated with reduced ejection fraction after myocardial infarction [20]. Lipopolysaccharide-binding protein (LBP) and soluble CD14 (sCD14) are acute-phase proteins that amplify LPS signaling and are widely used as surrogate markers of endotoxemia [21]. Intestinal fatty acid-binding protein (I-FABP) is a cytosolic protein specifically expressed in mature enterocytes; its elevated serum levels reflect enterocyte damage and loss of intestinal epithelial integrity, and have been shown to correlate with infarct size and worse cardiac function in acute heart failure [22]. Zonulin, a protein that modulates tight junction permeability, serves as another biomarker of increased intestinal permeability, with elevated circulating levels correlating with barrier disruption and predicting cardiovascular mortality [23,24]. Collectively, these biomarkers provide valuable tools for assessing gut barrier dysfunction in clinical settings.

The MAMP/PRR interaction exhibits multi-specificity. NOD1 recognizes antigens containing γ-D-glutamyl-meso-diaminopimelic acid (iE-DAP), which are present in bacterial peptidoglycan (PGN) from all Gram-negative and some Gram-positive bacteria. NOD2 recognizes muramyl dipeptide (MDP), a peptidoglycan motif derived from both Gram-negative and Gram-positive bacterial cell walls [25]. Additionally, NOD2 as a viral PRR can sense viral ssRNA to activate interferon production and antiviral defense [26]. The intestinal flora comprises a diverse collection of micro-organisms. Their cell wall components serve as ligands for Toll-like receptors (TLRs). For instance, lipopolysaccharide (LPS) from Gram-negative bacteria is the primary ligand for TLR4. Peptidoglycan and lipoteichoic acid (LTA) from Gram-positive bacteria are recognized by the TLR2/TLR1 heterodimer. Furthermore, TLR2 also recognizes lipopeptides from both Gram-positive and Gram-negative cell walls, as well as the Gram-positive surface proteins BP (lysozyme-resistant) and BSSP (lysozyme-sensitive). TLR3 recognizes double-stranded RNA (dsRNA), while TLR7 and TLR8 recognize single-stranded RNA (ssRNA). Additionally, mannan oligosaccharides and other polysaccharides from the fungal cell wall serve as ligands for TLR2 and TLR4.

Cardiomyocytes express a repertoire of TLRs (including TLR2, 3, 4, 5, 7, and 9) that enable them to sense pathogens and modulate their physiology. Ligation of TLR2, TLR4, and TLR5 triggers an NF-κB-mediated inflammatory response, characterized by the production of IL-6, chemokines (KC, MIP-2), and ICAM-1, which can significantly alter cardiac contractility [27]. While many microbial components drive inflammation, the gut microbiota presents a nuanced picture. A screen of 277 human gut bacterial strains showed that while phyla like *Actinobacteria* broadly stimulate TLR2, others like *Proteobacteria* are more selective for TLR4 [28]. Importantly, some commensals are protective. *Akkermansia muciniphila*, a *Verrucomicrobium*, has been identified as a key beneficial species. Both live and pasteurized forms of *A. muciniphila* attenuated infection-induced cardiac pathology, including LV hypertrophy and fibrosis. This cardioprotective effect is mechanistically linked to TLR2, as *A. muciniphila* was shown to activate AMPK and suppress the damaging NF-κB pathway while promoting anti-inflammatory IL-10 production in a TLR2-dependent manner.

The pathogenic effects of intestinal microbes can also be exerted indirectly through the translocation of their components into systemic circulation. In aged mice, compromised intestinal barrier integrity permits the translocation of microbial DNA from the lumen to distant cardiomyocytes, leading to an accumulation of bacterial DNA in the heart. Once inside cardiomyocytes, this leaked microbial DNA activates the cGAS/STING signaling pathway, upregulating the mRNA expression of pro-inflammatory mediators. This cascade induces myocardial inflammation and ultimately results in impaired cardiac function [29].

### 3.2. Microbial Metabolites Are Involved in Disease Development

The intestine possesses robust metabolic capabilities, with its resident microbiota participating in diverse enzymatic reactions that generate numerous metabolites [30]. These include short-chain fatty acids (SCFAs), free bile acids, trimethylamine/trimethylamine N-oxide (TMA/TMAO), indoxyl sulfate (IS), and phenylacetylglutamine (PAGln). These metabolites can exert local intestinal effects and systemically influence host physiology in both health and disease states. Current evidence indicates that myocarditis pathogenesis involves inflammatory responses, immune dysregulation, energy metabolism disturbances, and oxidative stress. Furthermore, cardiomyocyte hypertrophy, fibrosis, and apoptosis significantly impact disease prognosis in myocarditis patients. This review specifically examines the involvement of TMAO, SCFAs, and free bile acids in these pathological mechanisms.

#### 3.2.1. Derivatives of Intestinal Flora

The gut microbiota plays a pivotal role in converting dietary nutrients—such as choline, L-carnitine, and other trimethylamines from high-fat diets—into trimethylamine (TMA). This conversion is mediated by specific microbial enzyme systems, including the choline TMA–lyase complex (CutC/D) and the carnitine Rieske-type oxygenase/reductase systems (CntA/B and YeaW/X). After entering the portal circulation, TMA is oxidized to trimethylamine N-oxide (TMAO) by hepatic flavin-containing monooxygenase 3 (FMO3) [31]. The TMA/TMAO pathway is well-established as a contributor to atherosclerosis and adverse cardiovascular events. Mechanistically, TMAO upregulates the expression of CD36 and other scavenger receptors on macrophages, promoting cholesterol uptake and foam cell formation. Additionally, TMAO potentiates platelet activation by enhancing inositol trisphosphate (IP3) signaling, which increases intracellular Ca2+ release and leads to platelet hyper-reactivity and heightened thrombotic potential [31,32,33,34].

Short-chain fatty acids (SCFAs), comprising organic fatty acids with carbon chain lengths of 1–6 atoms (primarily acetate [C2], propionate [C3], and butyrate [C4]), represent crucial metabolites derived from microbial fermentation of dietary fibers and resistant starch within the intestinal lumen. The colon serves as the principal site of SCFA production, where these molecules are subsequently absorbed by colonic epithelial cells through proton-coupled (H+) or sodium-dependent monocarboxylate transporters. While partially metabolized locally, SCFAs enter systemic circulation where they function as metabolic substrates or signaling molecules [35,36]. SCFAs exert their pleiotropic effects through multiple mechanisms: (1) activation of G protein-coupled receptors (GPCRs) such as GPR41 and GPR43; (2) inhibition of histone deacetylases (HDACs); and (3) modulation of cellular metabolism via specific transporter proteins. It has been shown to modulate systemic immune function and energy metabolism, establishing them as key mediators of the gut–organ axis [36,37,38,39,40].

Bile acids (BAs) are cholesterol-derived metabolites synthesized in the liver as primary forms (CA, CDCA), conjugated, and secreted into the intestine to facilitate lipid absorption. While most are recycled via enterohepatic circulation, a fraction are metabolized by gut microbiota expressing bile salt hydrolase (BSH) into secondary bile acids (DCA, LCA, and UDCA). BSH enzymes are widely distributed among commensal bacteria, including Gram-positive genera (Clostridium, Bifidobacterium, Lactobacillus), Gram-negative Bacteroides species, and certain archaea [41]. Beyond their digestive functions, bile acids act as signaling molecules by activating nuclear (FXR, VDR, PXR) and G protein-coupled receptors (GPBAR1, M2/M3, S1PR2) [42,43].

The altered production of gut microbiota-derived metabolites in cardiomyopathy is associated with shifts in microbial community composition. In heart failure patients, SCFA-producing genera, including *Faecalibacterium*, *Roseburia*, and *Bifidobacterium*, have been reported to be reduced [44]. In murine viral myocarditis models, depletion of *Prevotellaceae_UCG-001* and *Odoribacter* has also been observed [45]. Concurrently, enrichment of *Clostridium_sensu_stricto* has been associated with elevated TMAO levels and myocarditis severity in immune checkpoint inhibitor-associated myocarditis models [46]. Regarding bile acids, gut dysbiosis has been shown to alter the abundance of BSH-harboring taxa (*Lactobacillus*, *Bifidobacterium*, *Clostridium*), which may shift the balance between primary and secondary bile acids [45].

#### 3.2.2. Potential Mechanisms for the Involvement of Intestinal Flora Derivatives in the Development of Myocarditis

##### Gut Microbiota-Derived Metabolites Significantly Modulate Inflammatory Responses in Host Physiology

Gut-derived metabolites exert different effects on inflammatory pathways relevant to cardiovascular pathology. Trimethylamine N-oxide (TMAO) directly promotes inflammation, as demonstrated in both chronic choline-fed and acute TMAO-injected mice. Mechanistically, TMAO activates the p38 MAPK/ERK/NF-κB cascade in vascular cells, inducing inflammatory markers, including COX-2, IL-6, E-selectin, and ICAM-1 [47]. Substantial evidence confirms that TMAO activates the NLRP3 inflammasome and caspase-1 both in vivo and in vitro, leading to cleavage of pro-IL-1β and pro-IL-18 into their active forms [48]. This process enhances susceptibility to atherosclerosis and other vascular pathologies by promoting cellular pyroptosis, altering membrane permeability, and inducing cellular dysfunction. The pro-inflammatory effects of TMAO-mediated NLRP3 activation have been established in multiple disease models [49], where it triggers NF-κB nuclear translocation, mitochondrial ROS generation, inflammasome assembly, and M1 macrophage polarization, as observed in murine models of graft-versus-host disease. Furthermore, TMAO potentiates TNF-α-induced inflammatory mediator release and stimulates fibroblast proliferation and collagen production via the Akt/mTOR/ERK signaling pathway [50].

In contrast, short-chain fatty acids (SCFAs) exhibit anti-inflammatory properties by reducing pro-inflammatory cytokines (TNF-α, IL-6, IFN-γ) while elevating anti-inflammatory IL-10 levels. SCFAs modulate immune responses by regulating T-cell differentiation and function, particularly through promoting regulatory T-cell (Treg) generation [40,51]. Butyrate, a prominent SCFA, acts as a potent histone deacetylase (HDAC) inhibitor that enhances histone acetylation, thereby suppressing expression of inflammation-related genes in immune cells. This HDAC-inhibitory property has been implicated in the anti-inflammatory effects of butyrate, suggesting a potential mechanism by which SCFAs may modulate inflammatory responses relevant to cardiac pathology [52].

Bile acids function as damage-associated molecular patterns (DAMPs) that synergize with ATP to promote calcium influx and mitochondrial redistribution, thereby activating the NLRP3 inflammasome [53]. The nuclear receptor FXR, which preferentially binds primary bile acids, demonstrates anti-inflammatory activity by suppressing the NF-κB-IL-1βaxis. Cardiac-expressed FXR also regulates apoptosis through mitochondrial death signaling pathways. The membrane receptor GPBAR1, which is essential for bile acid signaling, is widely expressed in cardiovascular tissues and immune cells. Recent studies identified GPBAR1 in cardiomyocytes, where its activation attenuates pro-inflammatory cytokine production (IL-1β, IL-6, TNF-α) and NF-κB expression in high glucose-challenged cardiomyocytes [54,55].

##### Gut Microbiota-Derived Metabolites Significantly Modulate Immune Responses in Myocarditis Pathogenesis

Histopathological evidence from endomyocardial biopsy (EMB) specimens reveals extensive immune cell infiltration, predominantly T lymphocytes and macrophages, within necrotic myocardial tissue [56]. Emerging evidence suggests that TMAO may exacerbate such autoimmune responses through multiple mechanisms that target these key immune cell populations. TMAO promotes pro-inflammatory Th17 cell differentiation and enhances B-cell maturation [57]. Experimental studies demonstrate that TMAO dose-dependently increases protein kinase C (PKC) activity in endothelial cells while promoting B-lymphocyte maturation. Furthermore, TMAO drives macrophage polarization toward the M1 phenotype through NLRP3 inflammasome activation, creating a cytokine milieu that favors differentiation of naïve CD4+ T cells into Th1 and Th17 subsets. Additionally, TMAO enhances antitumor immunity and induces pyroptosis via protein kinase R-like endoplasmic reticulum kinase (PERK) activation [49].

SCFAs represent a potent class of immunomodulators that regulate various immune cell populations, including regulatory T cells (Tregs), Th17 cells, regulatory B cells (Bregs), and macrophage subsets. On B cells, SCFAs—particularly butyrate—have been shown to enhance regulatory B cell (Breg) function and promote bacterial clearance, while simultaneously suppressing germinal center B cell and plasma cell differentiation. SCFAs also modulate B-cell activation and antibody production through inhibition of NF-κB and MAPK signaling pathways [58]. Butyrate and propionate exert HDAC inhibitory effects that directly influence gene expression in CD8+ cytotoxic T lymphocytes (CTLs) and Tc17 cells, upregulating IFN-γ and granzyme B expression while promoting Tc17-to-CTL phenotypic conversion [59].

The vitamin D receptor (VDR), a nuclear receptor for bile acids, plays crucial immunoregulatory roles in cardiac inflammation. Clinically, reduced VDR expression correlates with Th2-biased inflammation in myocarditis and advanced heart failure patients. VDR-deficient mice spontaneously develop myocarditis characterized by Th2-dominant inflammation, which can be ameliorated by VDR reconstitution [60]. Mechanistically, VDR forms complexes with GATA3 in CD4+ T cells from both human and murine hearts, thereby inhibiting IL-4 gene transcription and limiting Th2 cell differentiation—a potential therapeutic strategy for myocarditis.

##### Gut Microbiota-Derived Metabolites Significantly Influence Cardiac Energy Metabolism and Oxidative Stress Pathways in Myocarditis

Reactive oxygen species (ROS), which are intimately linked to both innate and adaptive immunity, exacerbate autoimmune processes in myocardial inflammation. The suppression of antioxidant defenses and sustained oxidative stress represent key pathological mechanisms driving cardiac remodeling in inflammatory cardiomyopathy [61]. While normal myocardial contraction relies on mitochondrial energy production, the dysregulated inflammatory milieu in myocarditis directly impairs mitochondrial function. The accumulation of damaged mitochondria severely compromises cellular energetics, elevates ROS generation, and activates innate immune responses, ultimately resulting in cardiomyocyte injury and contractile dysfunction [62].

Emerging evidence indicates that gut dysbiosis-associated disorders correlate with heightened oxidative stress. Modern dietary patterns and sedentary lifestyles disrupt microbial homeostasis, potentially leading to excessive ROS bioavailability and subsequent oxidative damage [63]. TMAO exacerbates these processes through direct binding and activation of protein kinase R-like endoplasmic reticulum kinase (PERK) or via endoplasmic reticulum (ER) stress-mediated NLRP3 inflammasome activation. These mechanisms critically contribute to apoptosis, inflammation, and the progression of cardiovascular and metabolic diseases.

In contrast, SCFAs demonstrate protective effects by elevating antioxidant enzymes while reducing serum malondialdehyde (MDA) and ROS concentrations [64]. SCFAs restore lipid oxidation balance through upregulation of antioxidant enzymes and effectively mitigate TNF-α-induced oxidative stress. Specific SCFA-producing bacteria, such as *Bifidobacterium longum subsp. longum YS108R*, exhibit dual anti-inflammatory and antioxidant properties through the distinct regulation of the NF-κB pathway and the nuclear factor erythroid 2-related factor 2 (Nrf2; encoded by the NFE2L2 gene) pathway, respectively [65].

The transcription factor Nrf2, a master regulator of the antioxidant defense system, maintains redox homeostasis by activating downstream effectors such as heme oxygenase-1 (HO-1). However, Nrf2 expression is downregulated in the cardiac tissue of myocarditis patients. This compromised antioxidant function accelerates pathological myocardial remodeling [66,67,68].

Bile acids exhibit concentration-dependent cardiac effects. While elevated levels demonstrate cardiotoxicity—particularly lipophilic species that induce mitochondrial dysfunction—they also activate beneficial pathways. Pathologically, increased bile acids suppress peroxisome proliferator-activated receptor gamma coactivator 1α (PGC-1α) expression, a master regulator of energy metabolism that influences cholesterol homeostasis, endothelial function, and oxidative stress responses. Conversely, bile acids exert protective effects through GPBAR1 receptor activation, which stimulates the Nrf2/HO-1 antioxidant pathway and enhances cardiac stress resistance via Akt, PKA, and ERK1/2 signaling cascades [54]. These mechanisms collectively improve exercise capacity and attenuate pathological cardiac remodeling.

##### Gut Microbiota-Derived Metabolites Significantly Influence Cardiomyocyte Hypertrophy and Fibrotic Remodeling in Myocarditis

The progression of myocardial fibrosis and cardiac dysfunction is driven by complex interactions between inflammatory and metabolic dysregulation. The gut-derived metabolite TMAO contributes to this pathology through multiple mechanisms. In cardiomyocytes, TMAO activates the TGF-β1/Smad3 pathway, inducing Smad3 phosphorylation and upregulating hypertrophic markers. Concurrently, it promotes cardiac fibroblast proliferation, migration, and collagen synthesis, with NLRP3 inflammasome activation and ROS generation further exacerbating fibrotic processes [69]. TMAO also disrupts cardiomyocyte architecture by inducing microtubule densification and aberrant trafficking of junctophilin-2 (JPH2), a protein critical for T-tubule integrity and calcium handling. This JPH2 dysregulation leads to T-tubule remodeling and impaired calcium cycling, ultimately compromising cardiac function [70].

BAs exert context-dependent effects on the heart [71]. Elevated serum BA levels are associated with cardiac dysfunction and promote myocardial fibrosis through NF-κB-mediated upregulation of VCAM-1 expression [72]. In contrast, ursodeoxycholic acid (UDCA) confers cardioprotection by activating the GPBAR1 receptor, stimulating cAMP release and inhibiting IL-11-induced myofibroblast transdifferentiation and extracellular matrix overproduction [73].

In addition to metabolite-driven pathways, evidence from a neonatal lactose intolerance (NLI) rat model suggests that gut-originating inflammatory signals may also contribute to long-term cardiac remodeling. In this model, NLI-induced systemic inflammation during early life was associated with cardiomyocyte hyperpolyploidy, transcriptomic rearrangements, and epigenetic alterations, including DNA and telomere instability and reactivation of the fetal gene program, culminating in left ventricular dilation and reduced ejection fraction in adulthood [74,75]. These observations indicate that gut-derived inflammatory insults during developmental windows can affect cardiac structure and function in the long term, complementing the metabolite-centric mechanisms discussed above and providing additional experimental support for the involvement of gut–heart signaling in cardiac remodeling.

##### Gut Microbiota-Derived Metabolites Influence Cardiomyocyte Apoptosis

Cardiomyocyte death is a critical contributor to cardiac dysfunction in myocarditis. Pathogens can activate multiple cell death pathways—including apoptosis, necrosis, and pyroptosis—thereby exacerbating disease progression.

SCFAs and their derivatives exert cardioprotection by counteracting pro-inflammatory cell death. Disruption of the PPARα and CYP4X1-mediated 14,15-EET-EA positive feedback loop in colonic macrophages upregulates pro-inflammatory cytokines (IL-1β, TNF-α), amplifying PD-1/PD-L1 inhibitor-associated cardiotoxicity. Butyrate, acting as a natural PPARα ligand, counteracts this process by preventing NF-κB-dependent M1 macrophage polarization and subsequent cardiomyocyte damage [76].

Bile acid signaling through FXR plays a dual role in regulating cardiac apoptosis. On one hand, FXR knockdown exacerbates diabetic cardiomyopathy, manifesting as myocardial disorganization, nuclear abnormalities, fibrosis, and cardiolipin accumulation, while FXR activation suppresses NF-κB signaling and reduces pro-inflammatory and pro-fibrotic factor production. On the other hand, studies by He et al. demonstrate that cardiomyocyte-specific FXR activation can trigger apoptosis through mitochondrial death pathways. Importantly, FXR silencing (via siRNA) or inhibition of mitochondrial signaling significantly reduce cardiomyocyte apoptosis. These findings position FXR as a novel and context-dependent regulator of cardiomyocyte apoptosis, with therapeutic potential evidenced by reduced infarct size and improved cardiac function following FXR inhibition.

To facilitate comparison of the evidence discussed above, we have summarized key gut microbiota–myocarditis studies in Table 1.

#### 3.2.3. Caveats and Evidence Gaps

While the experimental studies discussed above provide mechanistic insights into the roles of gut microbiota-derived metabolites in myocardial inflammation and remodeling, several important caveats should be acknowledged regarding the translational relevance of these findings. SCFAs are often described as beneficial, yet this generalization oversimplifies their biology. Depending on concentration and local tissue context, some SCFAs have been shown to promote neutrophil recruitment rather than suppress inflammation [78]. Clinical data directly linking any of these metabolites to myocarditis or cardiomyopathy are surprisingly sparse; most available evidence comes from heart failure cohorts or animal models [79]. TMAO levels, for instance, vary considerably with diet and renal function, which complicates the interpretation of observational studies [80]. A similar caveat applies to bile acids, where the reported cardiac effects are largely correlational and may be confounded by underlying liver or metabolic disease [81]. Taken together, although the gut–heart axis is an attractive concept, the current evidence base specific to cardiomyopathy remains largely preclinical. We have tried to reflect this limitation throughout the review.

## 4. Targeting the Gut Microbiota as a Novel Adjunctive Strategy to Improve Cardiovascular Health

**Figure 3:**
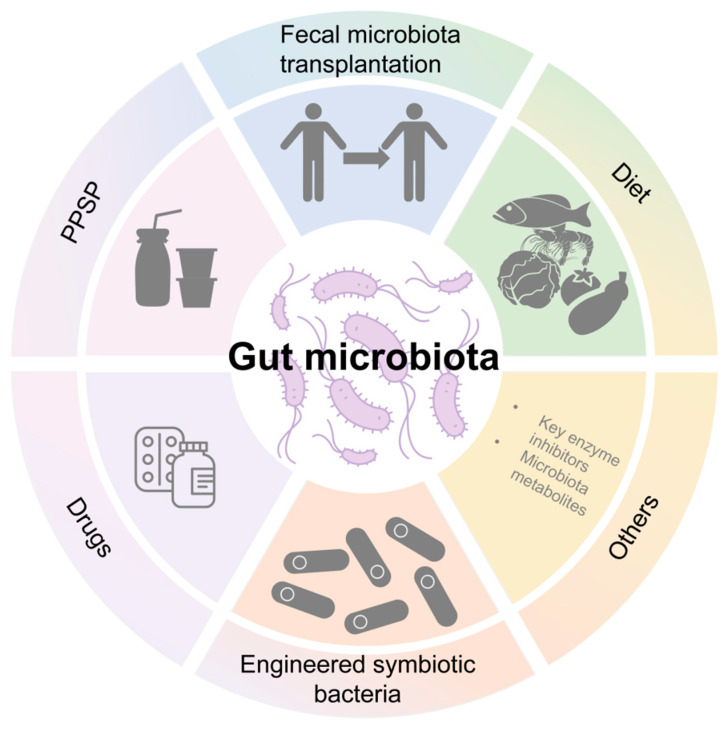
Strategies for modulating gut microbiota to improve cardiovascular diseases. The primary interventional strategies for modulating gut microbiota to improve cardiovascular diseases include fecal microbiota transplantation (FMT); probiotics, prebiotics, synbiotics, and postbiotics (PPSP); dietary modulation; drugs; genetically engineered bacteria; and other therapeutic approaches, such as inhibitors targeting key enzymes involved in microbiota-derived metabolite production. Figure 3 was generated using Microsoft PowerPoint.

Emerging therapeutic approaches targeting gut microbiota modulation show potential for cardiovascular disease management, particularly in myocarditis. Current strategies include fecal microbiota transplantation (FMT), probiotics, prebiotics, synbiotics, and antibiotic interventions (Figure 3). By ameliorating dysbiosis-associated pathophysiological processes, such interventions could potentially mitigate myocardial inflammation and improve clinical outcomes [82].

### 4.1. Fecal Microbiota Transplantation

Fecal microbiota transplantation (FMT)—the transfer of processed stool from healthy donors to recipients—has been documented in Chinese medical practice for millennia as a treatment for dysbiosis-related disorders through gut microbiome remodeling [83]. While clinical evidence in cardiovascular disease remains limited, preclinical studies have demonstrated promising therapeutic effects.

In experimental autoimmune myocarditis (EAM) models, FMT induces microbial compositional shifts characterized by increased *Bacteroidetes* and decreased *Firmicutes* abundance, correlating with improved myocardial histopathology and reduced inflammatory infiltration. Mechanistically, FMT is hypothesized to mediate therapeutic effects through three inter-related pathways: (1) restoration of microbial diversity and ecological balance; (2) immunomodulation via the gut–immune axis; and (3) attenuation of systemic inflammation. In doxorubicin-induced cardiotoxicity models, FMT ameliorated cardiac dysfunction by downregulating pro-fibrotic markers (TGF-β1, collagen I/III) and suppressing NOX-2/TLR-2-mediated oxidative stress while concurrently enhancing intestinal barrier integrity through upregulation of tight junction proteins (ZO-1) and reducing plasma endotoxin levels. Microbiome analysis revealed FMT’s capacity to reverse chemotherapy-induced dysbiosis, particularly through enrichment of beneficial taxa including *Prevotellaceae_UCG-001*, *Alloprevotella*, and *Rikenellaceae_RC9* [77,84].

Although there is a lack of evidence that microbiota transplantation improves major cardiovascular outcomes like myocardial infarction or death, early clinical studies have yielded preliminary results in areas such as hypertension and pulmonary arterial hypertension. A 2025 multicenter randomized double-blind trial demonstrated, for the first time, that oral fecal microbiota capsules are safe in hypertensive patients and significantly reduce systolic blood pressure within one week (by 4.34 mmHg compared to placebo). In a separate phase 1 trial in patients with pulmonary arterial hypertension, microbiota transplant therapy was also shown to be safe and feasible, with transient reductions in circulating pro-inflammatory cytokines [85,86].

Although generally considered safe when performed under standardized protocols, FMT carries potential risks contingent upon donor screening rigor, recipient immunocompetence, and administration methodology. Reported adverse events range from transient gastrointestinal symptoms (diarrhea, abdominal cramps) to severe complications such as bacteremia and sepsis, particularly in immunocompromised populations. Current evidence underscores the necessity for stringent donor selection criteria, standardized microbiota preparation processes, and comprehensive peri-procedural monitoring to mitigate risks.

### 4.2. Probiotics, Prebiotics, Synbiotics and Postbiotics (PPSPs)

Probiotics, prebiotics, synbiotics, and postbiotics (PPSPs) constitute emerging therapeutic agents for gut microbiota modulation, with demonstrated potential in cardiovascular management. Defined by the FAO/WHO as “live micro-organisms conferring health benefits when administered in adequate amounts,” probiotics function synergistically with prebiotics—non-digestible substrates (primarily oligosaccharides) that selectively stimulate beneficial bacterial growth [87].

Accumulating evidence supports the cardiovascular benefits of PPSPs. In patients with coronary heart disease, probiotics supplementation significantly reduces systemic inflammation and oxidative stress [88]. Randomized controlled trials in elderly patients with type 2 diabetes and high cardiovascular risk have demonstrated that synbiotics supplementation not only improves body weight and body fat but also significantly lowers low-density lipoprotein cholesterol, total cholesterol, fasting blood glucose, and insulin resistance, while reducing vascular cell adhesion molecule-1 (VCAM-1), a key marker of atherosclerosis [89]. A 2025 study found that *Bifidobacterium* supplementation in unstable angina patients improved gut microbiota and reduced trimethylamine N-oxide, a cardiovascular disease-related metabolite [90].

Experimental evidence supports strain-specific cardiovascular effects of certain probiotics. For instance, *Lactobacillus rhamnosus GR-1* has been shown to attenuate cardiomyocyte hypertrophy, although the underlying mechanisms remain unclear [91]. Additionally, the secreted metabolites of *Faecalibacterium prausnitzii*, a well-known butyrate-producing commensal, inhibit NF-κB activation and suppress IL-8 production in intestinal epithelial cells [92], suggesting that postbiotics may exert anti-inflammatory effects independently of live bacterial administration—a property that could be relevant to cardiac inflammatory conditions.

Emerging evidence highlights the potential of probiotics and prebiotics in combating antimicrobial resistance through gut microbiota modulation. A systematic meta-analysis revealed significant differential efficacy among microbial formulations: *Clostridioides difficile* demonstrated 82.4% decolonization rates with probiotic interventions, while *Lactobacillus* spp. and *Saccharomyces boulardii* achieved 71% and 77% pathogen clearance, respectively [93]. These agents may enhance colonization resistance through competitive exclusion or synergism with conventional antimicrobials. Prebiotics, while promoting commensal bacterial growth, require rigorous clinical evaluation to exclude potential pathogen-promoting effects—a critical consideration due to microbiota–nutrient specificity.

The therapeutic paradigm extends to combinatorial approaches, where probiotic co-administration counteracts antibiotic-induced dysbiosis, preserving microbial diversity and metabolic homeostasis. Synbiotics—rational combinations of probiotics and selectively utilized prebiotic substrates—demonstrate amplified efficacy through nutrient–microbe cross-feeding dynamics [94]. Contrastingly, postbiotics (non-viable microbial components/metabolites) offer advantages in stability and safety profiles compared to live probiotics, though their production standardization remains challenging [95,96].

### 4.3. Adjustment of Diets

Dietary patterns exert profound modulatory effects on gut microbiome composition and metabolic functionality, with emerging evidence highlighting their therapeutic potential in cardiovascular disease management [97]. The Mediterranean diet—recommended by authoritative guidelines including those of the European Society of Cardiology and the American Heart Association—significantly reduces the risks of major cardiovascular events, myocardial infarction, stroke, and cardiovascular death [98]. A 2025 umbrella review, analyzing data from over 17.15 million individuals, further reported strong evidence that high dietary fiber intake is associated with reduced cardiovascular disease mortality. These findings collectively underscore the importance of a plant-based, fiber-rich dietary pattern in cardiovascular health management [99].

Mechanistically, the Mediterranean diet stabilizes the gut microbiome through three inter-related pathways: (1) optimizing the *Firmicutes*/*Bacteroidetes* ratio; (2) enriching beneficial taxa (*Muribaculum*, *Rikenellaceae*, *Alloprevotella*); and (3) restoring mitochondrial function via upregulation of oxidative phosphorylation complexes (UQCRB, NDUFA2, ATP5PB), thereby attenuating LPS-induced inflammation [100]. The Green Mediterranean variant amplifies these benefits by driving *Prevotella* dominance and enhancing BCAA catabolism while suppressing BCAA biosynthesis [101].

High-fiber diets promote SCFA-producing bacteria (*Lactobacillus*, *Bifidobacterium*) and stimulate SCFA production, exerting anti-inflammatory effects. In contrast, Western diets (which are high-calorie, low-fiber) reduce microbial diversity and promote pro-inflammatory cytokine profiles [97]. Comparative analyses further reveal diet-specific immune modulation: vegan diets enhance innate immunity (antiviral responses, macrophage activation), whereas ketogenic diets boost adaptive immunity (T-cell proliferation, B-cell differentiation, NK cell enrichment) [102]. These findings highlight the therapeutic potential of dietary interventions, though interindividual variability in microbial engraftment necessitates personalized nutritional approaches.

### 4.4. Pharmaceutical Microbiomics

Emerging evidence indicates that pharmacological interventions can modulate the gut microbiota, thereby contributing to cardiovascular disease prevention and therapeutic strategies. This bidirectional interaction, termed pharmacomicrobiomics, exerts dual influences on both pharmacokinetic (PK) and pharmacodynamic (PD) processes [103]. The gut microbiota directly modulates drug metabolism through enzymatic regulation. Conversely, pharmacological agents reciprocally alter microbial ecology by disrupting metabolic homeostasis and growth dynamics of commensal species [104]. These reciprocal interactions establish a complex feedback loop between host drug response and microbial community structure, with significant implications for personalized therapeutic approaches.

Pharmacological agents exert profound effects on gut microbiota composition. The oral hypoglycemic agent metformin can significantly alter the intestinal flora structure in patients with coronary heart disease and, by regulating serum metabolites, confers long-term benefits, including a 5-year reduction in major adverse cardiovascular events [105]. Enalapril decreases circulating trimethylamine N-oxide (TMAO) levels via microbial trimethylamine lyase inhibition [106], whereas captopril modulates autonomic nervous system activity to restore microbial equilibrium. Furthermore, several medications, including proton pump inhibitors, metformin, and aspirin, have been shown to influence gut microbiota composition [107]. The combination of traditional Chinese medicine with probiotics also shows potential; for instance, Naoxintong Capsule (NXT) combined with statins exhibit superior lipid-lowering and anti-inflammatory effects compared to statins alone, an effect linked to regulation of specific intestinal bacteria [108].

Natural compounds targeting microbial metabolic pathways offer additional therapeutic avenues. Natural compounds such as apigenin, berberine, and quercetin alleviate experimental autoimmune myocarditis through upregulation of Th1 and Th2 cytokines and modulation of the MAPK signaling pathway [109]. Additionally, lupeol(Lup) ameliorates myocarditis by targeting the PPARα/LACC1/NF-κB axis to suppress macrophage M1 polarization and pyroptosis [110]. Notably, 3,3-dimethyl-1-butanol (DMB), a choline analog, inhibits microbial TMA synthesis and subsequent TMAO production, attenuating macrophage cholesteryl ester accumulation and atherosclerotic plaque progression in preclinical models [111].

### 4.5. Genetically Engineered Bacteria

Genetically engineered bacteria represent an emerging platform for targeted drug delivery [112]. Although no clinical studies have yet been completed on the use of genetically engineered bacteria to improve cardiovascular disease outcomes, preclinical animal experiments have yielded promising results. These studies target core pathological mechanisms through diverse strategies, including regulating lipid metabolism and inflammation via secretion of short-chain fatty acids or PCSK9 nanobodies [113,114]; intervening in antihypertensive pathways through expression of the ACE2-mimetic enzyme B38-CAP [115]; and delivering human ACE2 via genetically engineered *Lactobacillus casei* in the intestine to exert blood pressure-lowering effects through modulation of the local renin–angiotensin system [20].

Recent advances in synthetic biology have further expanded the therapeutic potential of this approach. A novel engineered gene regulation system, based on the synthetic inducer aTC, enables precise control of gene expression in commensal bacteria such as *Bacteroides* spp. within the gut without compromising microbial stability [116]. This system has proven effective across multiple *Bacteroides* species, including strains directly isolated from the human gut.

This technology establishes a foundation for engineering microbial communities to express cardiovascular protective factors (e.g., anti-inflammatory proteins or metabolic enzymes) and dynamically regulate microbial enzymatic activity. It paves the way for developing “smart microbiota” capable of sensing host biomarkers (e.g., inflammatory factors) and autonomously adjusting therapeutic gene expression in response to disease states.

## 5. Challenges and Future Directions in Targeting Gut Flora to Improve Myocarditis

Myocarditis treatment currently relies on conventional therapies to control inflammation and manage complications. While targeting the gut–cardiac axis has shown preclinical promise—with microbiota interventions restoring homeostasis and attenuating inflammation—clinical application remains nascent. Key unresolved questions—including strain-specific efficacy, intervention timing, and long-term safety—must be addressed through randomized controlled trials to establish these approaches as viable therapeutic options.

### 5.1. Limitations of Treatment Methods

The etiology of many myocarditis cases remains unclear, complicating the development of individualized treatment strategies. Existing therapeutic drugs often exhibit significant side effects, and some patients may progress to dilated cardiomyopathy or heart failure, resulting in poor prognoses. Thus, there is an urgent need to explore novel therapeutic approaches to enhance clinical outcomes.

Targeting the gut microbiota represents a promising avenue for improving myocarditis management, yet several challenges persist. The gut microbiome is a highly complex ecosystem comprising trillions of micro-organisms, with considerable interindividual variability. Despite growing evidence implicating gut microbial metabolites in myocarditis pathogenesis, identifying specific bacterial taxa or metabolites responsible for disease modulation remains difficult. Furthermore, interventions such as probiotics, fecal microbiota transplantation (FMT), and dietary modifications lack standardized protocols, and clinical trials in this area are limited. Safety and efficacy concerns also accompany these treatments. Nevertheless, these challenges present opportunities for further investigation into microbiome-based therapies for myocarditis.

### 5.2. Future Directions and Prospects

#### 5.2.1. Targeting Enterobacterial Enzymes Specific to the Disease Process

Dietary choline and its gut microbe-dependent metabolite, trimethylamine N-oxide (TMAO), can exacerbate the development of adverse ventricular remodeling and the fibrotic response during pressure overload. 3,3-Dimethyl-1-butanol (DMB), a natural inhibitor of TMA cleavage enzymes, may be an effective strategy for targeting the gut flora and thereby treating cardiovascular disease [111]. Administration of DMB to mice raised on a high-choline diet resulted in the observation of reduced circulating TMAO levels, foam cell formation, and atherosclerotic plaque reduction. These findings suggest that pharmacological modulation of TMAO biosynthesis and dietary interventions could mitigate cardiac dysfunction. Unlike conventional antibiotics, DMB inhibits microbial enzymes without promoting antibiotic resistance, highlighting its potential as a targeted therapy for cardiovascular disease.

#### 5.2.2. Maintain Beneficial Concentrations of Enterobacterial Metabolites

Short-chain fatty acids (SCFAs) demonstrate significant cardioprotective properties. Pham and colleagues developed a probiotic formulation (*EcN_TL*) that sustains physiological plasma SCFA concentrations while conferring myocardial protection. This probiotic blend exerts anti-inflammatory effects through suppression of the NF-κB signaling pathway. Furthermore, *EcN_TL* treatment reduces neutrophil infiltration in myocardial infarct regions and facilitates the polarization of macrophages toward a wound-healing phenotype [113].

#### 5.2.3. Improve the Efficiency of Drug Delivery

Oral nanomedicines represent a promising strategy for targeted modulation of gut microbiota and microbial metabolites. Tang et al. developed an intestinal-targeted protein nanomedicine by assembling *Akkermansia muciniphila*-derived membrane protein *Amuc-1100* (which enhances intestinal barrier function), fluorinated polyetherimide, and hydrolytically stable hyaluronan [117]. This formulation improved gut microbial composition by enriching *Lachnospiraceae* family bacteria—key producers of short-chain fatty acids (SCFAs)—leading to elevated butyrate and valerate levels. These changes restored splenic and cardiac lymphocyte homeostasis, normalized T- and B-cell ratios, and alleviated doxorubicin-induced cardiotoxicity.

In a complementary approach, deoxycholic acid-modified polyethyleneimine (DA-PEI) formed stable siRNA complexes via electrostatic/hydrophobic interactions, enhancing intracellular delivery. This system effectively suppressed RAGE expression in vitro and in vivo, reducing cardiomyocyte apoptosis, inflammatory cytokine release, and left ventricular remodeling in a myocardial infarction model [118]. Together, these strategies highlight the therapeutic potential of microbiota-targeted nanomedicines in cardiovascular diseases.

#### 5.2.4. Regulation of Microbial Communities

Beyond conventional interventions such as fecal microbiota transplantation (FMT), prebiotics, probiotics, dietary modifications, and pharmacological agents, phage therapy has emerged as a novel strategy for microbiome modulation. Bacteriophages, which constitute approximately 90% of the human virome, play a crucial role in shaping bacterial populations [119]. These viruses can be harnessed either for targeted antimicrobial effects or for precise manipulation of microbial communities, demonstrating significant therapeutic potential. For instance, PreforPro (Deerland Probiotics and Enzymes), a phage cocktail targeting Escherichia coli, has been evaluated in two placebo-controlled clinical trials to assess its safety and efficacy in improving gut health through bacterial composition modulation [120].

Despite these advances, direct applications of phage therapy for myocarditis treatment remain limited. In a pioneering study, Yang et al. developed an innovative approach by engineering an artificial microRNA (AmiR) targeting the 3’ untranslated region (3’UTR) of the coxsackievirus B3 (CVB3) genome. The researchers conjugated these AmiRs to folate-coupled bacteriophage packaging RNAs (pRNAs), enabling targeted delivery of therapeutic RNA interference (RNAi) agents against CVB3 infection [121]. This strategy not only demonstrates the potential of phage-based delivery systems but also opens new avenues for RNAi-based antiviral therapies in the treatment of viral myocarditis.

#### 5.2.5. Future Research Directions

Future research directions should focus on two key aspects. First, in-depth investigation into the association between gut microbiota and myocarditis is needed. Advanced multi-omics technologies including metagenomics and metabolomics should be employed to identify specific microbial taxa and metabolites associated with myocarditis, thereby providing a theoretical foundation for precise interventions. Second, optimization of intervention protocols such as fecal microbiota transplantation (FMT) requires urgent attention. Large-scale clinical trials, advanced studies of fecal metabolites, and animal model validation are necessary to elucidate the therapeutic mechanisms of FMT. Concurrently, research efforts should be strengthened in donor–recipient matching, preparation of microbial capsule formulations, and establishment of standardized efficacy evaluation systems.

Furthermore, clarifying the mechanistic pathways of gut microbiota-targeted interventions—including immune modulation, intestinal barrier enhancement, antioxidative stress effects, and anti-inflammatory responses—will provide valuable insights for developing novel therapeutic agents and identifying potential treatment targets.

### 5.3. Evidence Gaps and Challenges in Myocarditis Research

The clinical translation of novel therapies for myocarditis is hampered by significant methodological limitations and persistent research gaps. Methodologically, the field lacks well-designed, large-sample randomized controlled trials. Evidence from systematic reviews is sobering: only one RCT on IVIG therapy had been published by 2005, and 86.7% of cell therapy studies were rated as having “moderate” methodological quality with high bias risks in multiple domains [122,123]. Even the largest anti-inflammatory trial to date, ARAMIS, was constrained by heterogeneous subjects, a small sample size (*n* = 120), and a short intervention duration [124].

Beyond these trial design issues, fundamental gaps in our understanding of myocarditis persist. Poor concordance between animal models and human disease has resulted in the failure of therapies successful in preclinical studies (e.g., specific immunosuppressants) to translate to clinical benefit [125]. Few RCTs have stratified patients by underlying etiology—viral, immune checkpoint inhibitor-related, or autoimmune—to enable precision medicine approaches [122,126]. High-risk populations, such as those with cardiogenic shock requiring mechanical circulatory support, remain severely understudied due to recruitment challenges. Additionally, despite the widespread clinical use of cardiac magnetic resonance (CMR) for diagnosis, no RCTs have correlated its fine features (e.g., myocardial edema and fibrosis patterns) with long-term clinical outcomes, leaving imaging-guided prognostication without robust evidence.

Despite growing evidence, significant uncertainties remain in understanding the gut microbiota–myocarditis axis. Causality has yet to be established due to the scarcity of longitudinal cohort and intervention studies. The dose–effect relationships and tissue specificity of microbial metabolites remain to be elucidated. Moreover, most mechanistic studies are based on non-myocarditis models, necessitating validation in myocarditis-specific settings. Additionally, standardized intervention protocols and efficacy assessment systems for microbiota-targeted therapies are currently lacking.

## 6. Summary

Myocarditis is a disease characterized by inflammation of the myocardium that can affect people of all ages, and in severe cases, it can lead to heart failure, arrhythmia, and even sudden cardiac death, which can affect patients’ quality of life and even threaten their lives. Gut micro-organisms can affect the body’s inflammatory response, immune system, oxidative stress and energy metabolism, and their relationship with cardiovascular disease has become a hot research topic. It has been found that gut microbial diversity is reduced in patients with myocarditis, gut microbial metabolites can also play an important role in the pathogenesis of myocarditis, and imbalanced gut microbes are related to the development of myocarditis. Modulation of gut microbes by FMT, probiotics, prebiotics, dietary patterns, and drugs can provide new potential therapeutic strategies for myocarditis treatment. However, clinical studies on gut microbes and myocarditis are still insufficient, and the specific mechanism of action between the two has not been fully elucidated. Considering the differences between individuals and the safety of therapeutic modalities, the approach of regulating gut flora still faces many challenges and limitations in clinical application. Future research needs to address these issues in order to promote the development and improvement of new therapeutic approaches.

## Figures and Tables

**Table 1:** Summary of gut microbiota–myocarditis association studies.

Study Type	Model/Population	Key Taxa or Metabolites	Main Outcome	Ref
Mendelian randomization	420,000+ participants (633 myocarditis cases)	*Clostridia*, *Bilophila* (↓ risk); *Lactobacillales*, *Clostridium innocuum* group (↑ risk)	Identified 14 microbial taxa with causal relationships to myocarditis risk	[10]
Mendelian randomization	GWAS meta-analysis (18,340 individuals)	*Escherichia*/*Shigella*	1-unit increase in *Escherichia*/*Shigella* concentration elevates myocarditis risk by 38.1%	[11]
Preclinical study	EAM mice	Increased microbial diversity, ↑ F/B ratio	Gut dysbiosis correlates with autoimmune myocarditis severity	[12]
Preclinical study	Aged mice	Microbial DNA translocation	Compromised gut barrier permits microbial DNA translocation to cardiomyocytes → activates cGAS/STING → myocardial inflammation and cardiac dysfunction	[29]
Preclinical study	Chronic choline-fed/acute TMAO-injected mice	TMAO	Activates p38 MAPK/ERK/NF-κB → induces COX-2, IL-6, E-selectin, ICAM-1	[47]
Preclinical study	Multiple disease models (in vivo/in vitro)	TMAO	Activates NLRP3 inflammasome/caspase-1 → pyroptosis, M1 macrophage polarization	[48,49]
In vitro/Preclinical study	Neonatal mouse cardiomyocytes (in vitro); HG-induced injury model	Bile acids (TGR5/GPBAR1 activation)	TGR5 agonist INT-777 attenuates HG-induced pro-inflammatory cytokine expression, NF-κB activation, ROS production, and apoptosis in cardiomyocytes via Nrf2/HO-1 pathway	[54]
In vitro/Preclinical study	Neonatal rat cardiomyocytes (in vitro); adult mouse MI/R model (in vivo)	Bile acids (FXR activation)	FXR activation induces cardiomyocyte apoptosis via mitochondrial death pathway; FXR inhibition or genetic ablation reduces infarct size and improves cardiac function post-MI/R	[55]
Clinical + Preclinical	Myocarditis/HF patients + VDR−/− mice	VDR	VDR deficiency correlates with Th2 inflammation in patients; VDR−/− mice spontaneously develop Th2 myocarditis; VDR–GATA3 complex inhibits IL-4 transcription	[60]
Preclinical study	Doxorubicin-induced cardiotoxicity mouse model	FMT (enriched *Alloprevotella*, *Prevotellaceae*_UCG-001, *Rikenellaceae*_RC9_gut_group)	FMT improves LVEF and FS, decreases cardiac fibrosis, reduces gut damage (↓ goblet cell loss, ↓ ulcers, ↓ lymphocyte infiltration), ↑ ZO-1, ↓ plasma endotoxin, and restores gut microbial diversity	[77]

Arrows indicate the direction of change (↑: increase/upregulation; ↓: decrease/downregulation).

## Data Availability

No new data were created or analyzed in this study. Data sharing is not applicable to this article.

## Abbreviations

ACE2	Angiotensin-converting enzyme 2.
ATP5PB	ATP synthase peripheral stalk subunit b.
BAs	Bile acids.
BCAA	Branched-chain amino acids.
BSH	Bile salt hydrolase.
CA	Cholic acid.
CD	Crohn’s disease.
CDCA	Chenodeoxycholic acid.
CMR	Cardiac magnetic resonance.
CntA/B	Carnitine monooxygenase subunits A/B.
COX-2	Cyclooxygenase-2.
CTLs	Cytotoxic T lymphocytes.
CutC/D	Choline trimethylamine-lyase subunits C/D.
DAMPs	Damage-associated molecular patterns.
DA-PEI	Deoxycholic acid-polyethylenimine.
DCA	Deoxycholic acid.
DCM	Dilated cardiomyopathy.
dsDNA	Double-stranded DNA.
EAM	Experimental autoimmune myocarditis.
EMB	Endomyocardial biopsy.
ER	Endoplasmic reticulum.
FMO3	Flavin-containing monooxygenase 3.
FMT	Fecal microbiota transplantation.
FXR	Farnesoid X receptor.
GALT	Gut-associated lymphoid tissue.
GPBAR1	G protein-coupled bile acid receptor 1.
GPCRs	G protein-coupled receptors.
HDACs	Histone deacetylases.
HO-1	Heme oxygenase-1.
ICAM-1	Intercellular adhesion molecule-1.
iE-DAP	γ-D-glutamyl-meso-diaminopimelic acid.
IP3	Inositol trisphosphate.
IS	Indoxyl sulfate.
IVIG	Intravenous immunoglobulin.
JPH2	Junctophilin-2.
KC	Keratinocyte chemoattractant.
LCA	Lithocholic acid.
LPS	Lipopolysaccharide.
LTA	Lipoteichoic acid.
M2/M3	Muscarinic acetylcholine receptors M2 and M3.
MAMP	Microbe-associated molecular pattern.
MDA	Malondialdehyde.
MDP	Muramyl dipeptide.
MIP-2	Macrophage inflammatory protein-2.
NDUFA2	NADH:ubiquinone oxidoreductase subunit A2.
NLRs	NOD-like receptors.
NOD1	Nucleotide-binding oligomerization domain-containing protein 1.
NOD2	Nucleotide-binding oligomerization domain-containing protein 2.
Nrf2	Nuclear factor erythroid 2-related factor 2.
PAGIn	Phenylacetylglutamine.
PAMPs	Pathogen-associated molecular patterns.
PCSK9	Proprotein convertase subtilisin/kexin type 9.
PGC-1α	Peroxisome proliferator-activated receptor gamma coactivator 1-alpha.
PGN	Peptidoglycan.
PK	Pharmacokinetics.
PPARα	Peroxisome proliferator-activated receptor alpha.
PRRs	Pattern recognition receptors.
PXR	Pregnane X receptor.
RAGE	Receptor for advanced glycation end products.
RCT	Randomized controlled trial.
ROS	Reactive oxygen species.
S1PR2	Sphingosine-1-phosphate receptor 2.
SCFAs	Short-chain fatty acids.
ssDNA	Single-stranded DNA.
TLRs	Toll-like receptors.
TMA	Trimethylamine.
TMAO	Trimethylamine N-oxide.
UDCA	Ursodeoxycholic acid.
UQCRB	Ubiquinol–cytochrome c reductase binding protein.
VCAM-1	Vascular cell adhesion molecule-1.
VDR	Vitamin D receptor.
ZO-1	Zonula occludens-1.
